# Comprehensive analysis of molecular epidemiological characteristics of Morganella intermedius: a novel genospecies of Morganella morganii frequently isolated from environmental sources

**DOI:** 10.1099/mgen.0.001560

**Published:** 2025-12-08

**Authors:** Jiawei Chen, Yingchun Xu, Meng Xiao, Yali Liu

**Affiliations:** 1 Department of Laboratory Medicine, State Key Laboratory of Complex Severe and Rare Diseases, Peking Union Medical College Hospital, Chinese Academy of Medical Sciences and Peking Union Medical College, Beijing, PR China; 2 Graduate School, Peking Union Medical College, Chinese Academy of Medical Sciences, Beijing, PR China

**Keywords:** environment, epidemiology, flagellar-related genes, *Morganella intermedius*, *Morganella morganii*

## Abstract

We conducted the first molecular epidemiological analysis of *Morganella intermedius,* a novel genospecies of *Morganella morganii* frequently isolated from environmental sources. We identified specific advantages unique to *M. intermedius*, including the presence of more flagellar-related genes, which may enhance its environmental adaptability. Additionally, we explained the reduced number of antimicrobial resistance genes in *M. intermedius*, which could be attributed to its possession of more type II and type III restriction–modification systems compared to *M. morganii* and *Morganella sibonii*. Most importantly, we propose that environmental *M. intermedius* strains may contribute to clinical infections, and clinical strains showed the potential to acquire important antimicrobial resistance genes. This underscores the urgent need for increased clinical awareness and enhanced surveillance of this emerging genospecies.

**Impact Statement**

While *Morganella morganii* has traditionally been classified into two genospecies, our study focuses on the recently identified third genospecies *Morganella intermedius*. Comparative genomic analyses reveal that *M. intermedius* exhibits distinct evolutionary advantages, including an expanded gene repertoire and enhanced environmental adaptability. These findings necessitate an urgent reassessment of its pathogenic potential in clinical settings. Through integrated bioinformatics approaches, we systematically characterize its molecular epidemiology and evolutionary drivers, establishing a critical foundation for future surveillance of this emerging environmental pathogen and its implications for public health.

## Data Summary

All genomic data were obtained from the NCBI Genome database, with the corresponding accession numbers and detailed information provided in Supplementary Material (Table S1, available in the online version of this article).

## Introduction

*Morganella morganii* has traditionally been categorized into two subspecies, *M. morganii* subsp. *sibonii* and subsp. *morganii*, according to trehalose fermentation ability [1]. However, a novel genospecies, designated as *Morganella intermedius* or *Morganella chanii* (henceforth recommended to be uniformly referred to as *M. intermedius*), has been identified in recent studies [2,4]. Prior studies have shown that, like *Morganella sibonii*, *M. intermedius* also possesses the trehalose operon (with ∼88% sequence identity to that of *M. sibonii*) and demonstrates the capability to ferment trehalose [2], but its molecular epidemiological characteristics remain largely unclear. Consequently, more research is urgently needed to better understand this novel genospecies and its potential clinical or ecological implications.

## Methods

After quality control analysis, a total of 912 genomes of *M. morganii* from NCBI genome datasets as of June 2024 were retrieved for analysis. The pairwise average nucleotide identity (ANI) values of *M. morganii* genome sequences were calculated using pyANI (https://github.com/widdowquinn/pyani). Comparative genomic analysis among different genospecies was conducted using Roary [5] and Scoary [6]. The differentially identified genes were annotated and analysed using eggNOG-mapper [7] and further analysed for Kyoto Encyclopedia of Genes and Genomes (KEGG) pathway enrichment analysis with the clusterProfiler package in R [8]. The antibiotic resistome was characterized with abricate screened against the ResFinder database [9], and virulence-related genes were identified based on previous literature [2]. Based on the recombination-free core-genome SNPs, a maximum-likelihood phylogenetic tree was constructed using RAxML [10]. iTOL (v3.0) was used to annotate the tree with background information of the strain [11]. Easyfig (v2.2.2) was used to visualize the linear alignment of the genetic environment surrounding flagellar-related genes [12]. Restriction–modification (R–M) systems in *M. morganii* were identified using DefenseFinder (v2.0.1) [13].

## Results and discussion

**Fig. 1.**
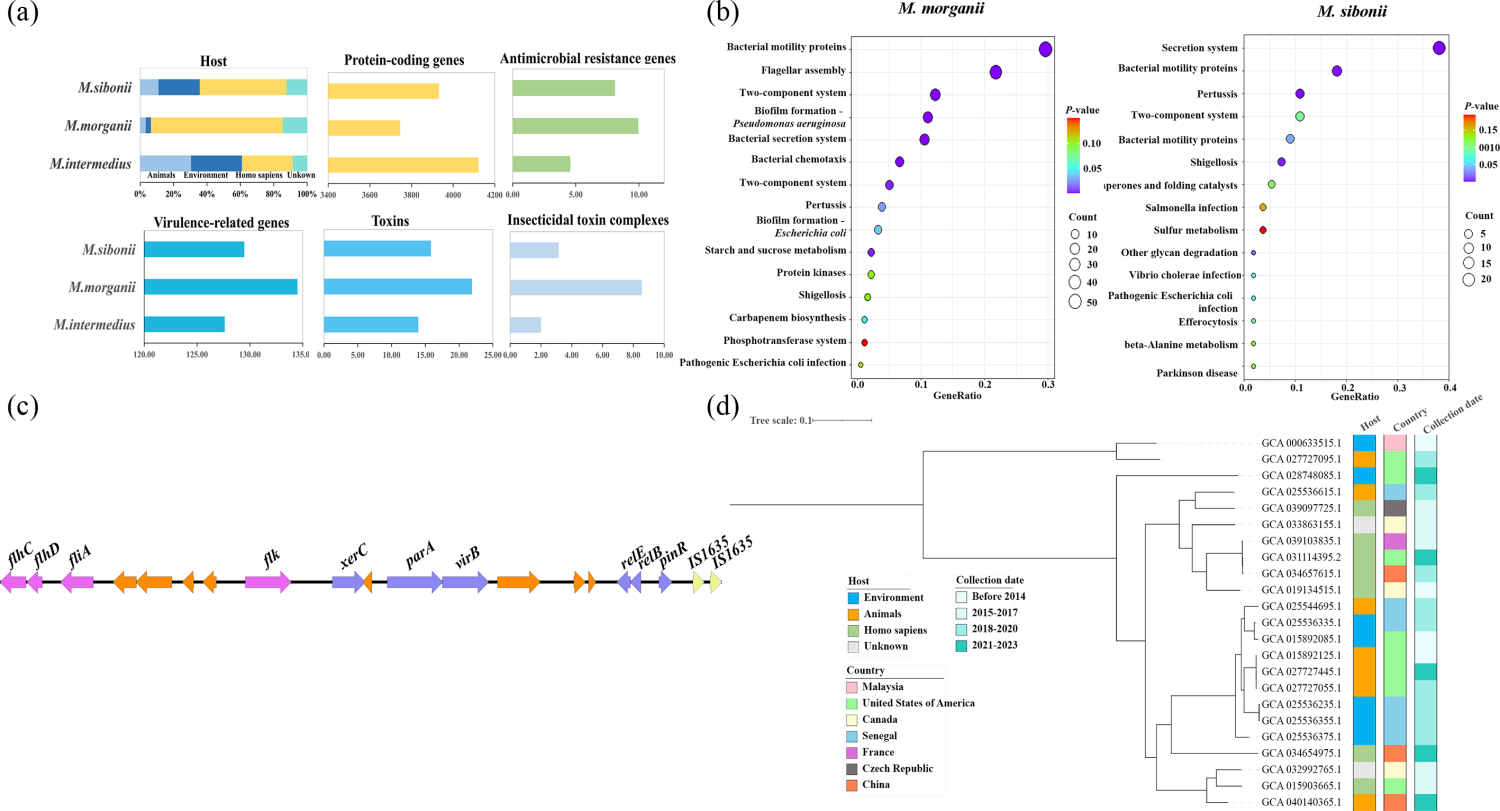
(a) Comparison of the epidemiological characteristics of the three genospecies of *M. morganii*. (b) KEGG enrichment analysis of unique genes in *M. intermedius* compared to *M. morganii* or *M. sibonii.* (c) The genetic context of flagellar-related genes within the genomic island in a representative isolate of *M. intermedius* (GCA_025536335.1). Purple arrows indicate flagellar-related genes, blue arrows represent genes with other functional roles, yellow arrows denote insertion sequences, and orange arrows correspond to hypothetical proteins. (d) The phylogenetic tree of 23 *M*. *intermedius* strains and corresponding background information.

In addition to *M. morganii* (814 isolates), *M. sibonii* (73 isolates) and *M. intermedius* (23 isolates), two additional genospecies were designated as *M. variant*1 (previously reported as *Morganella laugraudii* [3]) and *M. variant*2 (Table S1), exhibiting intra-genospecies ANI values greater than 95% (Fig. S1). *M. intermedius* strains demonstrated a broad geographic distribution, encompassing North America, Asia, Africa and Europe (Table S1, Fig. 1d). From a host perspective, unlike *M. morganii*, which was predominantly isolated from Homo sapiens, a significantly higher proportion of *M. intermedius* strains (30.43%) originated from environmental sources (Fig. 1a). Consistent with this environmental association, *M. intermedius* strains carried significantly fewer antimicrobial resistance genes (ARGs, 4.57 vs 9.94, *P*<0.05) and virulence-related genes (VRGs, 127.61 vs 134.49, *P*<0.05) compared to *M. morganii* (Fig. 1a). Specifically, compared to *M. morganii*, the reduction in ARGs among *M. intermedius* strains was evenly distributed across all types. In contrast, the significant reduction in VRGs was primarily concentrated within the insecticidal toxin complexes belonging to toxins, which is known for its potent insecticidal activity [14] (Fig. 1a).

To further investigate the potential reasons behind the lower prevalence of ARGs in *M. intermedius* strains, we profiled bacterial immune defence systems, which are known to influence horizontal gene transfer of ARGs via mobile genetic elements [15]. The results revealed that type II and type III R–M systems were uncommon in *M. morganii* (25.18% and 4.42%, respectively) and *M. sibonii* strains (24.66% and 5.48%, respectively) but were significantly overrepresented in *M. intermedius* strains (82.61% and 86.96%, respectively). With reference to the study by Yu Qiu *et al*.[16], further analysis of the association between type II and type III R–M systems and drug resistance revealed that strains carrying these systems harbour fewer ARGs compared to those without them (Fig. S2). Thus, type II and type III R–M systems may act as potential factors restraining the dissemination of ARGs in *M. intermedius* strains.

Interestingly, despite *M. intermedius* strains harbouring the fewest ARGs and VRGs, they exhibited the highest number of protein-coding genes compared to *M. morganii* (3,745.77) and *M. sibonii* strains (3,931.52), with an average of 4,121.87 (Fig. 1a). Comparative genomics revealed that among the unique genes of *M. intermedius* compared to *M. sibonii* and *M. morganii*, there was a significant presence of flagellar-related genes. KEGG enrichment analysis further indicated that these unique genes were predominantly associated with secretion systems, bacterial motility proteins and flagellar assembly (Fig. 1b), suggesting a potential for enhanced environmental adaptability. Notably, further analysis revealed that many of these flagellar-related genes were located within genomic islands (Fig. 1c), indicating that horizontal transfer via genomic islands has facilitated the integration of functional genes into *M. intermedius*, driving its evolution.

Phylogenetic reconstruction revealed two major clusters among M. intermedius strains, showing no strong correlation with host type, geographic origin or isolation time (Fig. 1d). Strains within the same cluster exhibited a broad host range, including animals, Homo sapiens and environmental sources. This indicated a strong connection between environmental and clinical strains, suggesting that environmental *M. intermedius* strains may contribute to clinical infections. Notably, among the seven clinical strains analysed, two clinical *M. intermedius* strains (GCA_031114395.2 and GCA_039103835.1) were found to carry multiple ARGs, including carbapenemase genes (*bla*_NDM-7_ and *bla*_NDM-1_, respectively), demonstrating that clinical *M. intermedius* strains have the capacity to acquire important ARGs. Furthermore, the presence of abundant flagellar-related proteins may enhance the adaptability of *M. intermedius* in clinical hosts, underscoring the need for increased clinical awareness and surveillance of this species.

In summary, this study identifies a novel genospecies, *M. intermedius*, within *M. morganii*, whose emergence may be linked to environmental stress coupled with horizontal transfer of genomic islands. Future clinical attention should be directed towards monitoring the evolution and dissemination dynamics of this genospecies.

## Supplementary material

10.1099/mgen.0.001560 Uncited Supplementary Material 1.

10.1099/mgen.0.001560 Uncited Table S1.

## Abbreviations

ANI: average nucleotide identity
ARGs: antimicrobial resistance genes
KEGG: Kyoto Encyclopedia of Genes and Genomes
R–M: restriction–modification
VRGs: virulence-related genes
