# Supplementary material for: Comprehensive analysis of molecular epidemiological characteristics of Morganella intermedius: a novel genospecies of Morganella morganii frequently isolated from environmental sources
Source: Microb Genom. 2025 Dec 8;11(12):001560. doi: 10.1099/mgen.0.001560 (PMC12684994; doi:10.1099/mgen.0.001560)
Supplement: Uncited Supplementary Material 1. [file mgen-11-01560-s001.pdf]

Supplementary Materials for

Comprehensive analysis of molecular epidemiological characteristics of *Morganella intermedius*: a novel genospecies of *M. morganii* frequently isolated from environmental sources

Jiawei Chen *et al.*

*Corresponding author. Email: liuyluijk@163.com.

This file includes:

Supplementary Methods and Supplementary Figs. S1 to S2.

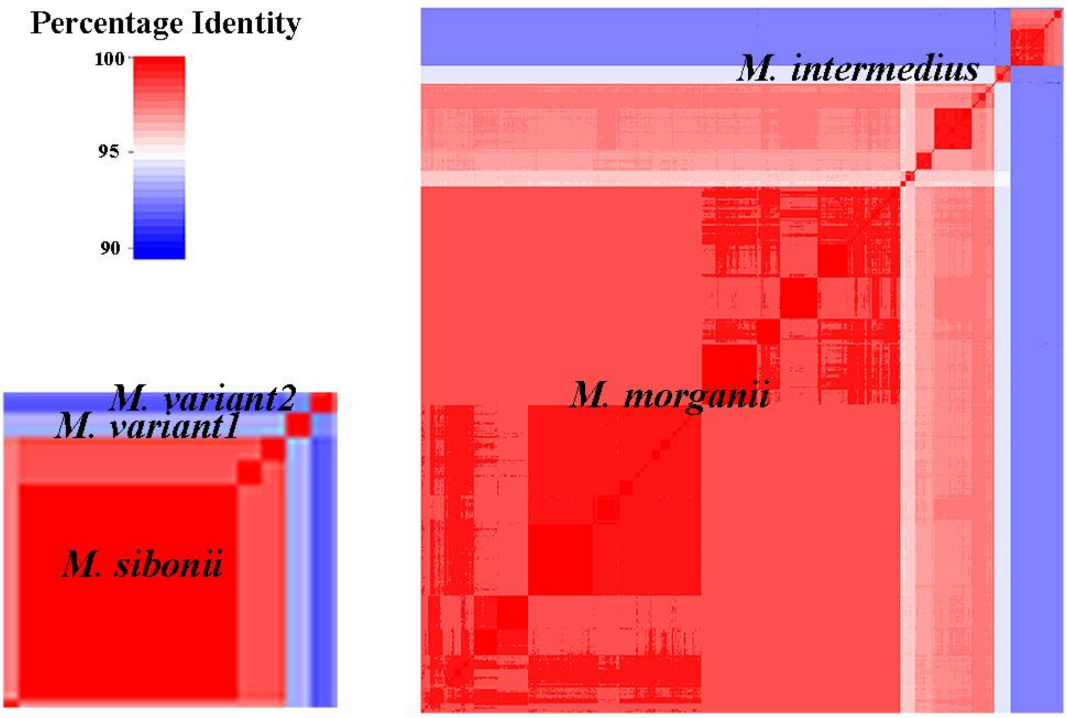

Figure S1. A pairwise average nucleotide identity (ANI) comparison was calculated for 912 *M. morganii* isolates, displayed on a heatmap where blue indicates low nucleotide identity and red indicates high nucleotide identity. The left diagram is an enlarged section of the right diagram.

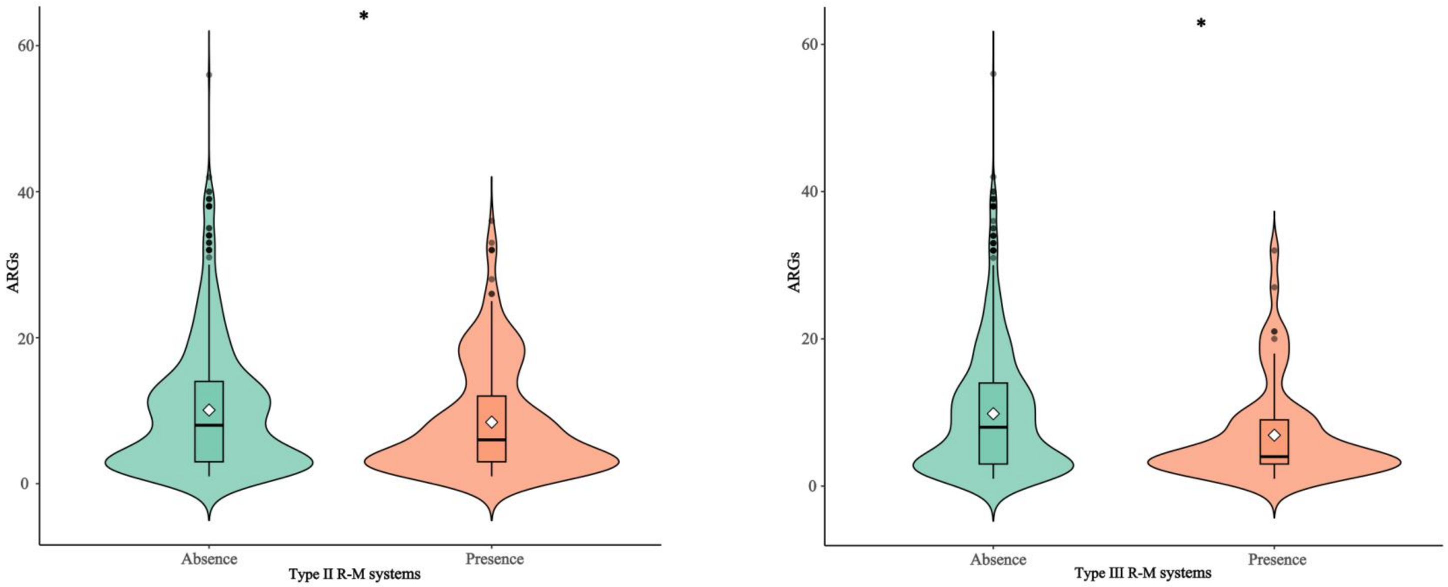

Figure S2. Comparison of the number of antimicrobial resistance genes in Morganella morganii with and without type II and type III restriction-modification (R-M) systems.
